# Overexpression of the Novel MATE Fluoroquinolone Efflux Pump FepA in *Listeria monocytogenes* Is Driven by Inactivation of Its Local Repressor FepR

**DOI:** 10.1371/journal.pone.0106340

**Published:** 2014-09-04

**Authors:** François Guérin, Marc Galimand, Fabrice Tuambilangana, Patrice Courvalin, Vincent Cattoir

**Affiliations:** 1 CHU de Caen, Service de Microbiologie, F-14033 Caen, France; 2 Université de Caen Basse-Normandie, EA4655 (équipe “Antibio-résistance”), F-14032 Caen, France; 3 Institut Pasteur, Unité des Agents Antibactériens, Paris, France; Arizona State University, United States of America

## Abstract

Whereas fluoroquinolone resistance mainly results from target modifications in gram-positive bacteria, it is primarily due to active efflux in *Listeria monocytogenes*. The aim of this study was to dissect a novel molecular mechanism of fluoroquinolone resistance in this important human pathogen. Isogenic *L. monocytogenes* clinical isolates BM4715 and BM4716, respectively susceptible and resistant to fluoroquinolones, were studied. MICs of norfloxacin and ciprofloxacin were determined in the presence or in the absence of reserpine (10 mg/L). Strain BM4715 was susceptible to norfloxacin (MIC, 4 mg/L) and ciprofloxacin (MIC, 0.5 mg/L) whereas BM4716 was highly resistant to both drugs (MICs 128 and 32 mg/L, respectively). Reserpine was responsible for a 16-fold decrease in both norfloxacin and ciprofloxacin MICs against BM4716 suggesting efflux associated resistance. Whole-genome sequencing of the strains followed by comparative genomic analysis revealed a single point mutation in the gene for a transcriptional regulator, designated *fepR* (for fluoroquinolone efflux protein regulator) belonging to the TetR family. The frame-shift mutation was responsible for the introduction of a premature stop codon resulting in an inactive truncated protein. Just downstream from *fepR*, the structural gene for an efflux pump of the MATE family (named FepA) was identified. Gene expression was quantified by qRT-PCR and demonstrated that *fepA* expression was more than 64-fold higher in BM4716 than in BM4715. The clean deletion of the *fepR* gene from BM4715 was responsible for an overexpression of *fepA* with resistance to norfloxacin and ciprofloxacin, confirming the role of FepR as a local repressor of *fepA*. In conclusion, we demonstrated that overexpression of the new MATE efflux pump FepA is responsible for fluoroquinolone resistance in *L. monocytogenes* and secondary to inactivation of the FepR repressor.

## Introduction

*Listeria monocytogenes* is a gram-positive rod-shaped facultative intracellular food-borne pathogen widely distributed in the environment [1]. It is responsible for severe human infections (such as bacteremia and central nervous system [CNS] infections) primarily in neonates, elderly people and patients with impaired cellular immunity, as well as abortions in pregnant women [2]. The reference treatment of listeriosis currently relies on a synergistic combination of high-dose ampicillin or amoxicillin and gentamicin administered intravenously [3]. Nonetheless, despite an effective therapy, CNS infections are associated with a high mortality rate (ca. 30%) while sequels are common [4].

Except for intrinsic resistance to cephalosporins and fosfomycin, *L. monocytogenes* is susceptible to all antibiotics in vitro, including fluoroquinolones (FQs). However, FQs are not recommended in the treatment of listeriosis even if newer compounds (i.e. levofloxacin, moxifloxacin) might represent an efficient alternative in the treatment of CNS listeriosis, as previously reported in one human case report [5] and several animal models [6], [7]. Due to the extensive use of FQs for the treatment of multiple infections, there is also an important selective pressure for recovery of in vivo FQ-resistant mutants in gram-positive bacteria including *L. monocytogenes*.

In gram-positive bacteria, FQ resistance is chromosomally encoded and most commonly results from the accumulation of mutations in molecular targets that are type II topoisomerases, DNA gyrase (GyrA_2_GyrB_2_) and topoisomerase IV (ParC_2_ParE_2_) [8]. Alterations predominantly occur within short conserved regions of the *gyrA*, *gyrB*, *parC*, and *parE* genes, the so-called quinolone-resistance determining regions (QRDRs). In contrast, FQ resistance in *L. monocytogenes* seems to be primarily due to active efflux, especially through overexpression of the *lde* gene coding for a transporter belonging to the major facilitator superfamily (MFS) [9]–[12].

In this study, we have elucidated a novel molecular mechanism of FQ resistance in a clinical isolate of *L. monocytogenes* (a preliminary report of this work was presented at the 53^rd^ Interscience Conference on Antimicrobial Agents and Chemotherapy, Denver, CO, 10–13 September 2013 [abstract C1-1440]).

## Materials and Methods

### Bacterial strains and molecular typing

Strains BM4715 and BM4716 (both belonging to the serotype 1/2b) were collected in France from a unique blood sample of the same patient suffering from listeriosis. Strain BM4715 was fully susceptible to ciprofloxacin whereas BM4716 was resistant. Reference strain *L. monocytogenes* EGD-e (serotype 1/2a) [13] was included for molecular typing. *Staphylococcus aureus* ATCC 29213, *S. aureus* SA-1199B (NorA-overproducing) [14], and *Escherichia coli* EC1000 (Life Technologies) were used as controls for antimicrobial susceptibility testing, efflux, and subcloning, respectively.

Strains BM4715 and BM4716 were typed by pulsed-field gel electrophoresis (PFGE) using the SmaI endonuclease, as previously described [15]. The PFGE patterns were analyzed in accordance with Tenover et al. [16].

### Antimicrobial susceptibility testing

MICs of antimicrobial agents (except for sparfloxacin and levofloxacin) were determined on Mueller-Hinton agar using E-test strips (bioMérieux, Marcy l’Etoile, France) with or without reserpine (10 mg/L). MICs of sparfloxacin and levofloxacin as well as those of antiseptics and dyes were determined on Muller-Hinton by the agar dilution method (tested range, from 0.06 to 256 mg/L) with or without reserpine (10 mg/L) with 10^4^ CFU per spot after 24 h of incubation at 35°C [17]. Determination of MICs was performed in three independent experiments.

### PCR amplification and sequencing

Genomic DNA from *L. monocytogenes* BM4715 and BM4716 was extracted using the QIAmp DNA Mini kit (Qiagen). QRDRs of *gyrA*, *gyrB*, *parC*, and *parE* genes were amplified by PCR with specific primers (Table S1) [10] and the purified PCR products were sequenced with the same sets of primers in both directions (GATC Biotech, Konstanz, Germany).

### Whole-genome sequencing

Genomic DNA was extracted from mid-log phase cultures of *L. monocytogenes* BM4715 and BM4716 using the NucleoBond buffer set III and the NucleoBond AX-G 100 (Macherey-Nagel, Hoerdt, France) following the manufacturer’s instructions. High-throughput sequencing was performed using an Illumina MiSeq Benchtop sequencer (ProfileXpert-LCMT, Lyon, France). The shotgun sequencing for *L. monocytogenes* BM4715 led to an assembly of 93 contigs sizing from 203 to 320,858 bp with an aggregate genome size of 2,984,196 bp and a 145.3X average coverage of the genome while data for BM4716 were as follows: 89 contigs sizing from 203 to 476,158 bp, aggregate genome of 2,984,010 bp, and a 150.6X average genomic coverage. Comparative genomic analysis was performed using the CLC Genomics Workbench software 6.5.1 (CLC bio, Aarhus, Denmark). The nucleotide and deduced amino acid sequences for each contig were analyzed with BlastN and BlastX programs available over the Internet at the National Center for Biotechnology Information website (http://blast.ncbi.nlm.nih.gov/Blast.cgi).

### RNA manipulations

Total RNA was extracted from BM4715 and BM4716 using the ZR Fungal/Bacterial RNA Miniprep kit (Zymo Research, Irvine, CA). Residual chromosomal DNA was removed by treating samples with the TURBO DNA-*free* kit (Life Technologies, Saint Aubin, France). Samples were quantified using the Biospec-Nano spectrophotometer (Shimadzu, Noisiel, France) and the integrity was assessed using the Agilent 2100 bioanalyzer.

For RT-PCR experiments, cDNA was synthesized from total RNA (∼1 µg) using the QuantiTect Reverse Transcription kit (Qiagen, Courtaboeuf, France) according to the manufacturer’s instructions. For operon mapping, PCR reactions were then carried out according to standard conditions using specific primers synthesized by Sigma-Aldrich France (Table S1). Each PCR amplification was performed on cDNA and chromosomal DNA (used as positive control). Transcript levels of the *fepA* gene were determined by the DeltaDelta Ct method and the *bglA* gene [18] was used as a housekeeping control gene (Table S1). Each experiment was performed in triplicate.

The transcription start site (TSS) and promoter sequences were determined using the 5′RACE System kit (Life Technologies SAS, Saint Aubin, France) using specific primers (Table S1) according to the manufacturer’s instructions.

### Construction of a BM4715 *fepR* deletion mutant

A fepR deletion mutant was derived from L. monocytogenes BM4715 (named BM4715ΔfepR) by allelic exchange with a truncated copy of fepR using the pWS3 suicide vector as previously described [19]. Approximately 500-bp fragments upstream and downstream from fepR were amplified by PCR using BM4715 chromosome as template and primer pairs Lmo-fepR-F1-EcoRI/Lmo-fepR-R1 and Lmo-fepR-F2/Lmo-fepR-R2-EcoRI (Table S1). Following EcoRI restriction, ligation and amplification using Lmo-fepR-F1-EcoRI and Lmo-fepR-R2-EcoRI, the resulting fragment carrying the truncated fepR copy was cloned in the temperature-sensitive pG(+)host9-derived shuttle vector pWS3 to create plasmid pWS3ΩfepR-KO. The hybrid plasmid was then introduced into the chromosome of BM4715 by electro-transformation and homologous recombination followed by excision of the wild-type copy as described [19]. Deletion of the fepR gene was confirmed by PCR and sequencing.

### Multiple alignment and phylogenetic analysis

Sequence comparison and phylogenetic analysis was performed by the neighbor-joining algorithm with the ClustalX software (version 1.83) and the resulting tree was displayed with TreeView software (version 1.6.6).

### Nucleotide sequence accession numbers

The nucleotide sequences of the *fepR*/*fepA* locus from strains BM4715 and BM4716 have been deposited in the GenBank database under accession no. KJ000253 and KJ000254, respectively.

### Ethics statement

Ethical approval was not required for the study since there was no direct patient involvement and only bacterial strains were retrospectively studied. In addition, clinical samples were de-identified and no identifiable patient information is available.

## Results

### Efflux-mediated FQ resistance in BM4716

**Figure 1:**
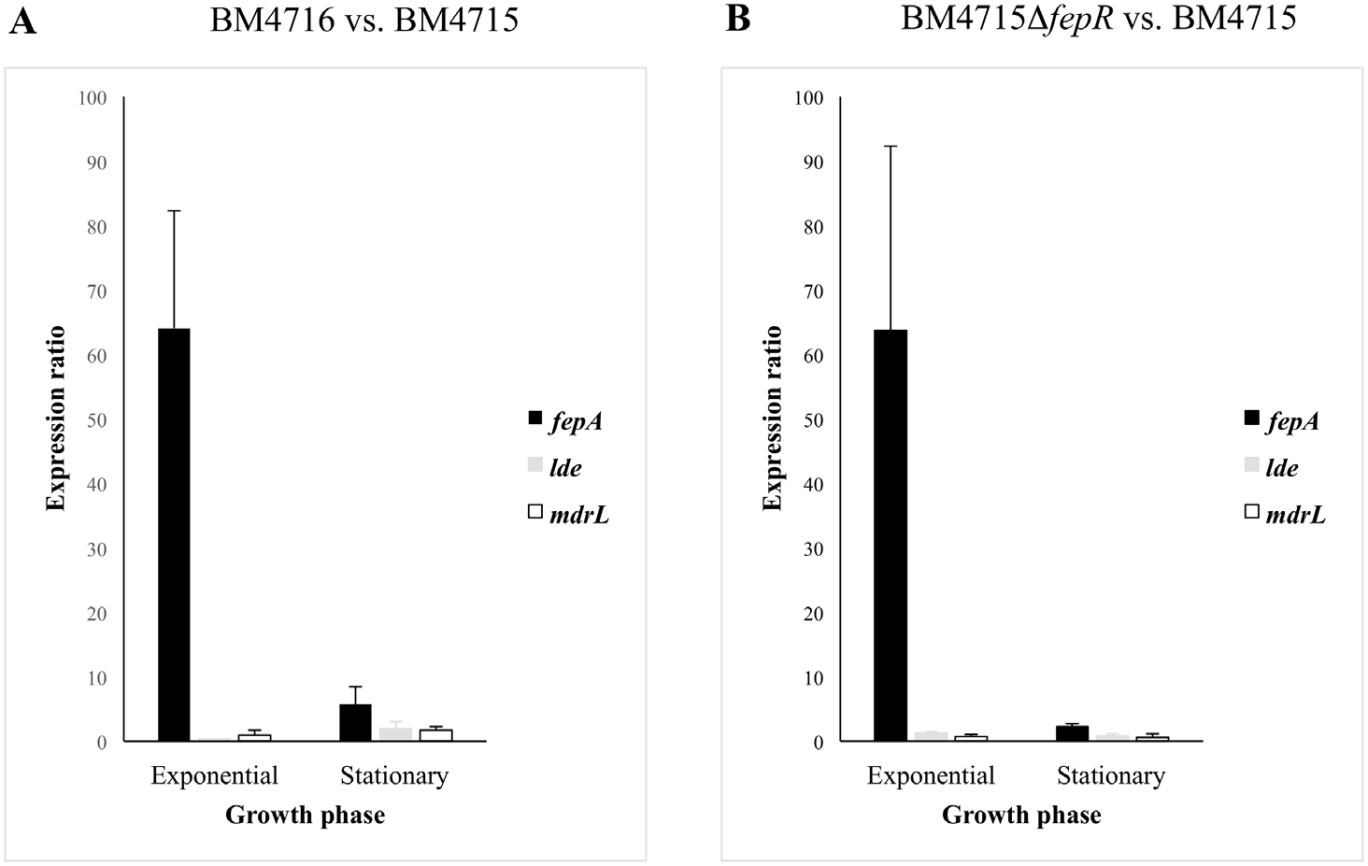
Expression ratios of the *fepA* gene in *L. monocytogenes* according to the bacterial growth phase. BM4716 vs. BM4715 (A), and *fepR* deletion mutant (BM4715Δ*fepR*) vs. BM4715 (B).

Comparison of antibiotic susceptibility profiles showed that strain BM4715 was susceptible to norfloxacin and ciprofloxacin (MICs of 4 and 0.5 mg/L, respectively) whereas BM4716 was resistant (MICs of 128 and 32 mg/L, respectively) (Table 1). Besides these two FQs, no significant differences in MICs between the two isolates were observed for other antibiotics (Table 1). However, few changes were noted for some antiseptics and dyes, such as cetylperidinium chloride (4-fold), chlorehexidine (4-fold), and ethidium bromide (8-fold) (Table 1). PFGE analysis confirmed that these strains (isolated in a unique clinical specimen from the same patient) were isogenic, revealing that BM4716 was a FQ-resistant mutant derived from BM4715 (Figure S1). QRDRs of *gyrA*, *gyrB*, *parC*, and *parE* genes were sequenced but no mutations were found, suggesting another mechanism of FQ resistance. Since norfloxacin and ciprofloxacin are hydrophilic FQs and well-known substrates of efflux pumps, we determined their MICs with or without reserpine. There was a significant change (16-fold decrease) in MICs against BM4716 in the presence of the efflux pump inhibitor, confirming efflux-related resistance (Table 1). Expression levels of *lde* and *mdrL* genes (both coding for efflux pumps known to be associated with fluoroquinolone resistance in *L. monocytogenes*) were not significantly different between BM4716 and BM4715 strains (Figure 1A), suggesting the implication of other(s) transporter(s).

**Table 1:** MIC of antibiotics, antiseptics, and dyes against *L. monocytogenes* clinical isolates BM4715 and BM4716 as well as BM4715 *fepR* deletion mutant (BM4715Δ*fepR*).

Compound^a^	MIC (mg/L)^b^					
	BM4715		BM4716		BM4715Δ*fepR*	
	−R	+R	−R	+R	−R	+R
**FQ antibiotic**						
**Norfloxacin**	4	2 (2)	128	8 (**16**)	64	4 (**16**)
**Ciprofloxacin**	0.5	0.5 (1)	32	2 (**16**)	16	1 (**16**)
**Sparfloxacin**	1	1 (1)	2	2 (1)	2	2 (1)
**Levofloxacin**	1	1 (1)	2	1 (2)	2	1 (2)
**Moxifloxacin**	0.25	0.25 (1)	0.5	0.25 (2)	0.5	0.25 (2)
**Non-FQ antibiotic**						
**Amoxicillin**	0.25	0.25 (1)	0.25	0.25 (1)	0.25	0.25 (1)
**Cephalothin**	2	2 (1)	2	2 (1)	2	2 (1)
**Gentamicin**	0.5	0.5 (1)	1	1 (1)	1	1 (1)
**Erythromycin**	0.25	0.25 (1)	0.25	0.25 (1)	0.25	0.25 (1)
**Clindamycin**	2	2 (1)	2	2 (1)	2	2 (1)
**Chloramphenicol**	4	4 (1)	4	4 (1)	4	4 (1)
**Tetracycline**	0.5	0.25 (2)	0.5	0.25 (2)	0.25	0.25 (1)
**Tigecycline**	0.12	0.12 (1)	0.12	0.06 (2)	0.06	0.06 (1)
**Vancomycin**	1	1 (1)	1	1 (1)	1	1 (1)
**Linezolid**	2	2 (1)	2	2 (1)	2	2 (1)
**Daptomycin**	1	0.5 (2)	1	0.5 (2)	1	0.5 (2)
**Cotrimoxazole**	0.03	0.03 (1)	0.06	0.03 (2)	0.03	0.03 (1)
**Rifampin**	0.06	0.06 (1)	0.06	0.06 (1)	0.06	0.06 (1)
**Fusidic acid**	2	2 (1)	2	2 (1)	2	2 (1)
**Fosfomycin**	≥1,024	≥1,024 (1)	≥1,024	≥1,024 (1)	≥1,024	≥1,024 (1)
**Antiseptic**						
**Benzalkonium chloride**	4	2 (2)	8	8 (1)	8	8 (1)
**Cetylperidinium chloride**	2	2 (1)	8	4 (2)	8	4 (2)
**Chlorhexidine**	2	2 (1)	8	8 (1)	8	8 (1)
**Tetraphenylphosphonium**	128	64 (2)	256	128 (2)	128	64 (2)
**Dye**						
**Acridine orange**	256	128 (2)	256	128 (2)	256	128 (2)
**Acriflavine**	64	64 (1)	128	128 (1)	64	64 (1)
**Crystal violet**	4	4 (1)	8	8 (1)	4	4 (1)
**Ethidium bromide**	32	16 (2)	256	128 (2)	256	128 (2)
**Rhodamine**	16	8 (2)	16	8 (2)	16	8 (2)

a FQ, fluoroquinolone.

b MICs determined in the presence (+R) or absence (−R) of reserpine (10 mg/L). Values in parentheses indicate the *n*-fold decrease in MIC in the presence of reserpin compared to its absence. Values in bold indicate significant changes in MIC.

### Single mutation within a *tetR*-like gene in BM4716

**Figure 2:**
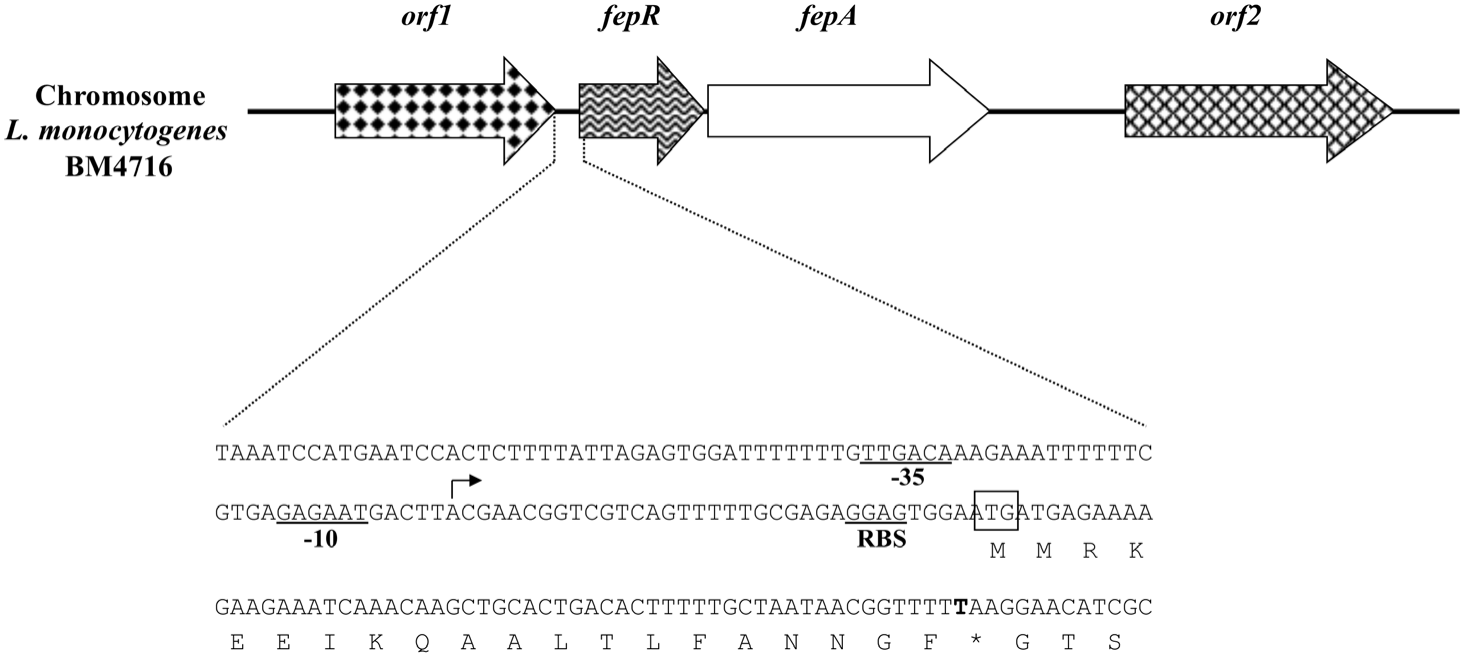
Schematic map of the genetic environment of *fepR*/*fepA* in *L. monocytogenes* BM4716 chromosome. Open reading frames (ORFs) are indicated by horizontal arrows. Genes *orf1* and *orf2* putatively encode a lipase and a DNA-binding protein, respectively. The sequence corresponding to the upstream region of *fepR*/*fepA* genes is presented in details. The −35 and −10 promoter boxes are underlined and the transcription start site (TSS) is represented by an arrow. The start codon of *fepR* and its putative ribosome-binding site (RBS) are indicated. The non-synonymous mutation G61T (leading to substitution E21*) is shown in bold.

**Figure 3:**
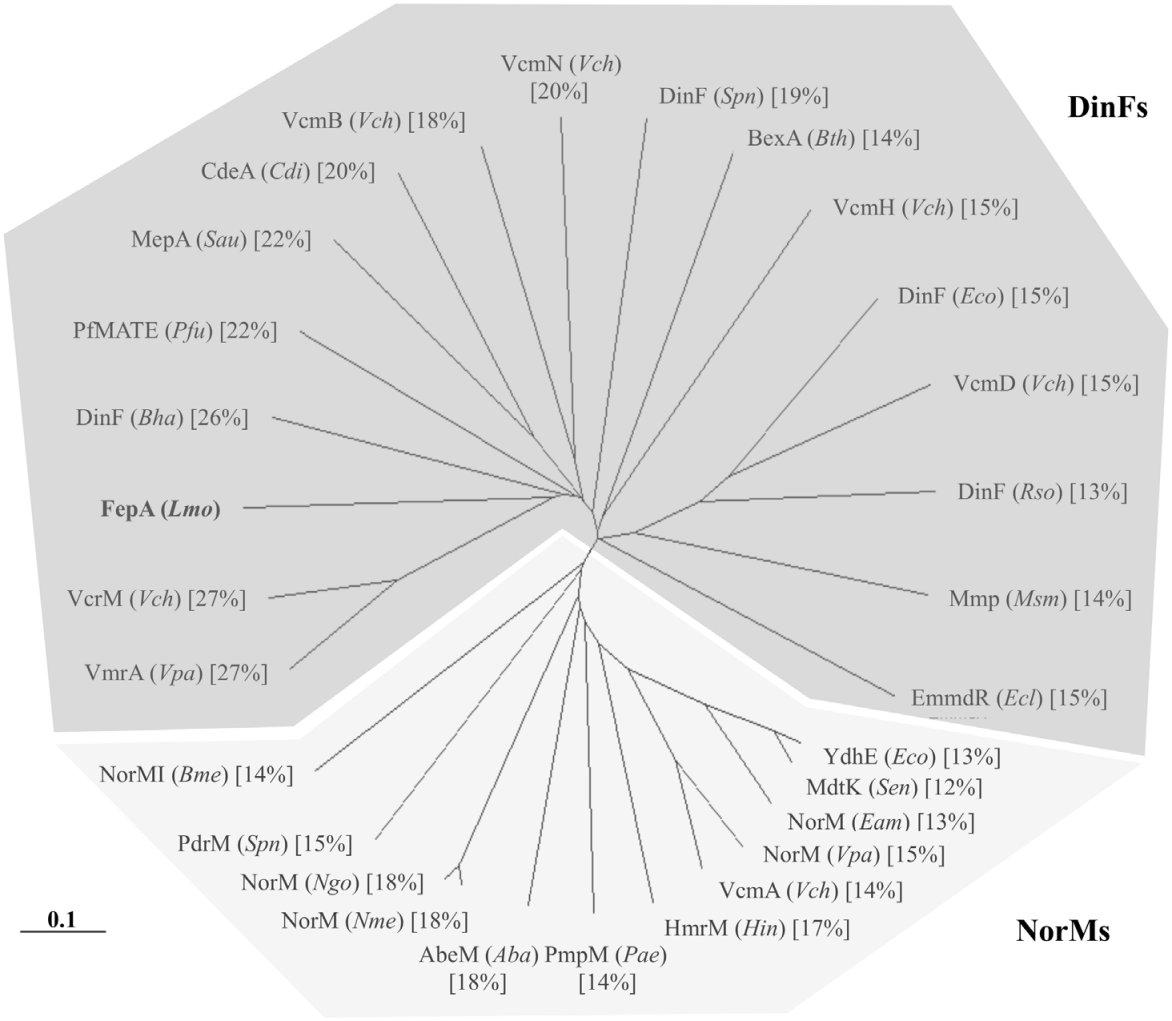
Phylogenetic tree based on neighbor-joining analysis of sequences of bacterial efflux proteins belonging to the MATE family. The various homologs were identified in: *Aba*, *Acinetobacter baumannii*; *Bha*, *Bacillus halodurans*; *Bme*, *Brucella melitensis*; *Bth*, *Bacteroides thetaiotaomicron*; *Cdi*, *Clostridium difficile*; *Eam*, *Erwinia amylovora*; *Ecl*, *Enterobacter cloacae*; *Eco*, *Escherichia coli*; *Hin*, *Haemophilus influenzae*; *Lmo*, *Listeria monocytogenes*; *Msm*, *Mycobacterium smegmatis*; *Ngo*, *Neisseria gonorrhoeae*; *Nme*, *Neisseria meningitidis*; *Pae*, *Pseudomonas aeruginosa*; *Pfu*, *Pyrococcus furiosus*; *Rso*, *Ralstonia solanacearum*; *Sen*, *Salmonella enterica* serovar Typhimurium; *Sau*, *Staphylococcus aureus*; *Spn*, *Streptococcus pneumoniae*; *Vch*, *Vibrio cholerae*; and *Vpa*, *Vibrio parahaemolyticus*. The scale bar represents 10% difference in amino acid sequences. Amino acid identities of each MATE protein as compared to FepA are indicated in square brackets. The two DinF and NorM subfamilies are highlighted.

By comparing the entire genome *L. monocytogenes* BM4716 to that of BM4715, we found only three mutations, including two silent mutations in the same gene (corresponding to *lmo0460* in *L. monocytogenes* EGD-e) coding for a membrane-associated lipoprotein and, most importantly, a single mutation within a 594-bp gene (corresponding to *lmo2088* in *L. monocytogenes* EGD-e) coding for a 197-amino-acid TetR-like transcriptional regulator. This mutation (G61T) was responsible for the occurrence of a premature stop codon (E21*) leading to a nonfunctional truncated protein (Figure 1). Immediately downstream from this gene, a 1,332-bp gene (corresponding to *lmo2087* in *L. monocytogenes* EGD-e) was identified, which coded for a 443-amino-acid efflux pump of the MATE family (Figure 2). Interestingly, this protein only shared 12% to 27% identity with other bacterial MATE efflux pumps (Figure 3).

### Overexpression of a MATE efflux pump in BM4716

Assuming that the efflux-mediated FQ resistance was likely due to this novel MATE efflux pump, we assessed the expression of its corresponding gene by qRT-PCR. The gene was highly overexpressed in BM4716 as compared to BM4715 in exponential phase (64-fold increase), while expression alteration was moderate in stationary phase (6-fold increase) (Figure 1A). The role of the *tetR*-like gene as repressor of *fepA* was confirmed by construction of the clean deletion mutant. Indeed, the strain BM4715Δ*fepR* was resistant to norfloxacin and ciprofloxacin (MICs of 64 and 16 mg/L, respectively) as observed with the BM4716 clinical isolate while there was a significant change (16-fold decrease) in MICs against BM4715Δ*fepR* in the presence of reserpine (Table 1). Also, there was an overexpression of *fepA* in BM4715Δ*fepR* as compared to BM4715 in exponential phase (64-fold increase) as observed in BM4716 (Figure 1B). The gene for the MATE efflux pump was named FepA (for fluoroquinolone efflux protein A) and that for the TetR-like transcriptional regulator designated FepR (for fluoroquinolone efflux protein regulator).

### Description of the *fepRA* operon

**Figure 4:**
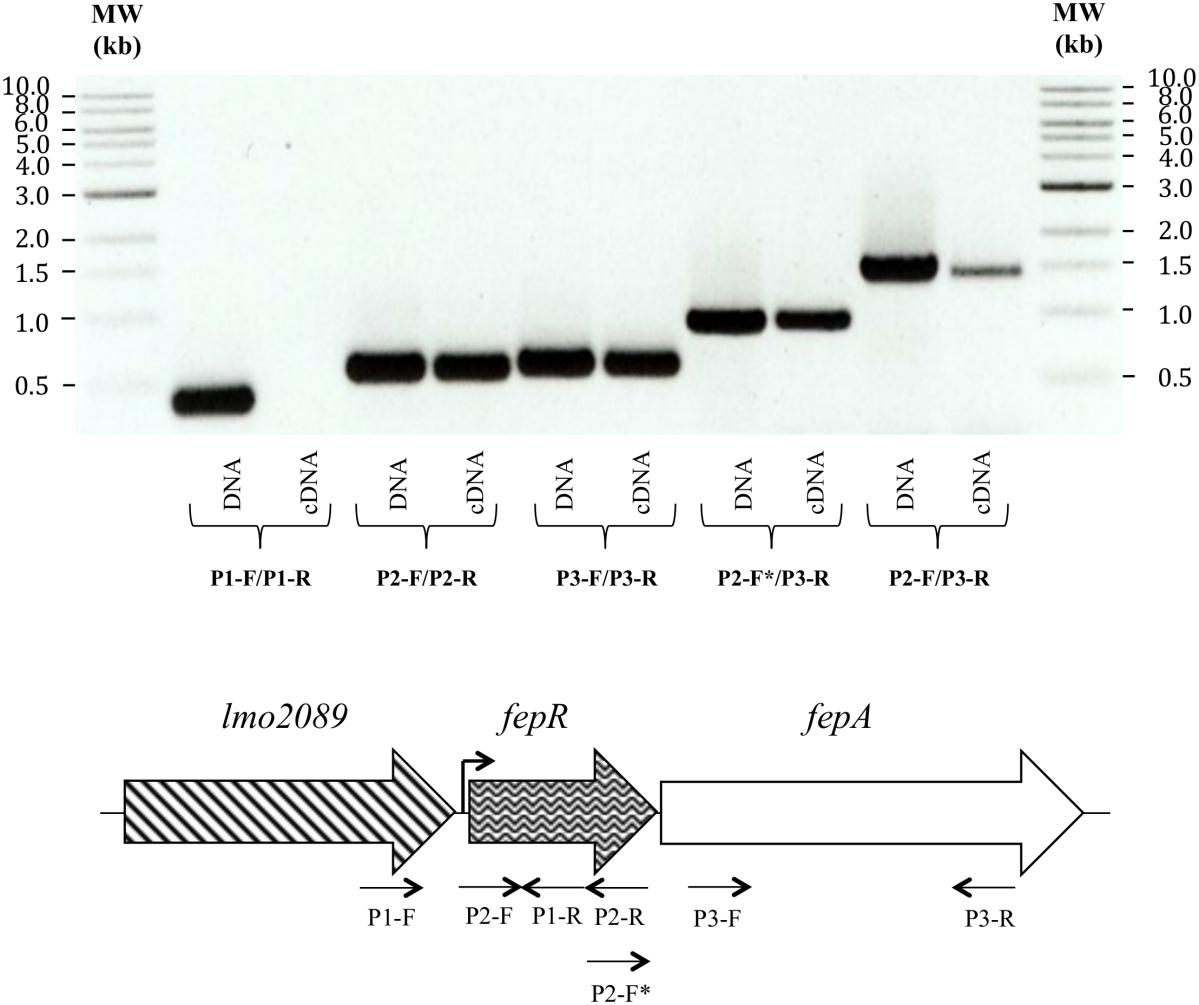
Agarose gel electrophoresis showing PCR products corresponding to transcripts of *fepR* and *fepA* genes. Different sets of primers were designed to amplify specific regions of *fepR* (P2-F/P2-R) or *fepA* (P3-F/P3-R), the intergenic region (P2-F*/P3-R), the long cotranscript (P2-F/P3-R) and a negative control (P1-F/P1-R) (Table S1). Each PCR amplification was carried out on chromosomal DNA (used as positive control) and on cDNA, as indicated. MW, 1-kb ladder (New England Biolabs, France).

Since an operon structure was bioinformatically predicted for the *fepA* and *fepR* genes (only 9 intervening bp), this was confirmed by RT-PCR (Figure 4). In addition, we experimentally determined a unique TSS 34 bp uptstream from the start codon of *fepR* (Figure 1). The *fepRA* locus was surrounded by two genes (according to *L. monocytogenes* EGD-e numbering): *lmo2089* (upstream) and *lmo2086* (downstream) coding for a lipase (347 amino acids) and a DNA-binding protein (423 amino acids), respectively (Figure 2).

## Discussion

Whereas FQ resistance in gram-positive bacteria mainly occurs through point mutations in QRDRs in ParC and GyrA, it has so far only been related to active efflux in *L. monocytogenes* [9]–[12]. There are five families of drug efflux systems: the major facilitator superfamily (MFS), the resistance-nodulation-cell division (RND), the small multidrug resistance (SMR), the multidrug and toxic-compound extrusion (MATE), and the ATP-binding cassette (ABC) family [20]. The first four catalyze drug extrusion by exploiting the transmembrane electrochemical (H^+^ or Na^+^) gradient whereas ABC transporters are powered by ATP hydrolysis. In gram-positive bacteria, active efflux is mainly associated with overexpression of MFS pumps, such as NorA in *S. aureus* and PmrA in *Streptococcus pneumoniae*, which preferentially extrude hydrophilic FQs (i.e. norfloxacin, ciprofloxacin) [21].

In *L. monocytogenes*, only two chromosomal pumps, both belonging to the MFS, have been involved in antimicrobial resistance [10], [22]. The first transporter, encoded by the *mdrL* gene, is related to the efflux of macrolides, cefotaxime, and heavy metals [22] while the second, encoded by the *lde* gene, is associated with resistance to hydrophilic FQs as well as with acriflavine and ethidium bromide resistance [10]. Hence, this is the characterization of the third antimicrobial efflux pump in *L. monocytogenes*. Even though the prevalence of FepA-mediated FQ resistance is not known, it might be not so rare since 13 out of 15 ciprofloxacin-resistant foodborne isolates of *L. monocytogenes* did not show overexpression of the *lde* gene, suggesting the existence of other resistance mechanisms [11].

Transporters of the MATE family have been rarely demonstrated to be involved in antimicrobial resistance [21]. However, almost all known MATE transporters can recognize FQs as substrates while acriflavine and ethidium bromide can also be pumped out [23]. The prototype of this family is NorM from *Vibrio parahaemolyticus* and its homolog in *E. coli* is YdhE [24], [25]. All members of the MATE family possess 12 transmembrane domains and usually function as Na+/drug antiporters. By phylogenetic analysis, MATE transporters are divided in three clusters. The first and the third cluster include homologs of NorM and DinF, respectively, whereas the members of the second cluster are exclusively found in eukaryotes [25]. FepA appears to be related to DinF homologs even if the degrees of identity are low.

In gram-positive bacteria, only four MATE members have been described: CdeA in *Clostridium difficile*, MepA in *S. aureus*, and DinF and PdrM in *S. pneumoniae* [26]–[30]. CdeA is able to confer FQ resistance in *E. coli* when overexpressed and resistance to acriflavin and ethidium bromide is also observed [26]. MepA has a broad substrate profile including biocides, FQs (norfloxacin, ciprofloxacin), and tigecycline [27], [28]. In *S. pneumoniae*, DinF is involved in FQ resistance while PdrM confers resistance to norfloxacin, acriflavine, and 4′,6-diamidino-2-phenylindole (DAPI) [29], [30]. Taken together, all MATE-family proteins described so far are able to extrude FQ agents in gram-positive bacteria.

Little is known about regulation of genes encoding MATE-family proteins in gram-positive bacteria. Only the transcriptional regulation of the *mepA* gene has been extensively studied. More specifically, it has been shown that it was controlled by a local MarR-type repressor called MepR [27]. In this work, we have demonstrated that *fepA* was also negatively controlled, but by a TetR-type repressor. TetR proteins constitute a well-known family of transcriptional repressors [31]. They have been extensively studied in the regulation of several genes for drug efflux systems, such as TetR and *tetA* in *E. coli*, AcrR and *acrAB* in *E. coli*, AdeN and AdeIJK in *Acinetobacter baumannii*, or QacR and *qacA*/*qacB* in *S. aureus* [32], [33]. As previously reported for other TetR-like repressors, FepR also autoregulates expression of its own gene [31]. As opposed to what was observed for *fepR*, TetR-like-encoding genes are usually divergently transcribed and are not part of an operon with the structural gene for the efflux pump [31]. Finally, no data are available about the expression of efflux pumps during the cell cycle. For *fepA*, it seems to be highly expressed during the exponential phase, like most of the genes controlled by σ^70^ factors, but further investigations are needed.

In conclusion, this is the first characterization of a MATE efflux pump involved in FQ resistance in *L. monocytogenes*. The substrate profile appears to be narrow, including only hydrophilic FQs. Finally, we also report transcriptional regulation of the expression of a MATE family efflux pump-encoding gene through a TetR-like repressor. Similar molecular mechanisms may be involved in FQ resistance within other important gram-positive pathogens in which FepA homologs are chromosomally encoded and for which FQ are indicated.

## Supporting Information

Figure S1 **PFGE patterns of SmaI-digested genomic DNA of** ***L. monocytogenes*** **strains. Lanes: 1, BM4715; 2, BM4716; 3, EGD-e.**
(TIF)

Table S1 **Deoxynucleotide primers used in the study.**
(DOCX)
