# Supplementary figures and images for: Overexpression of the Novel MATE Fluoroquinolone Efflux Pump FepA in Listeria monocytogenes Is Driven by Inactivation of Its Local Repressor FepR
Source: PLoS One. 2014 Sep 4;9(9):e106340. doi: 10.1371/journal.pone.0106340 (PMC4154695; doi:10.1371/journal.pone.0106340)

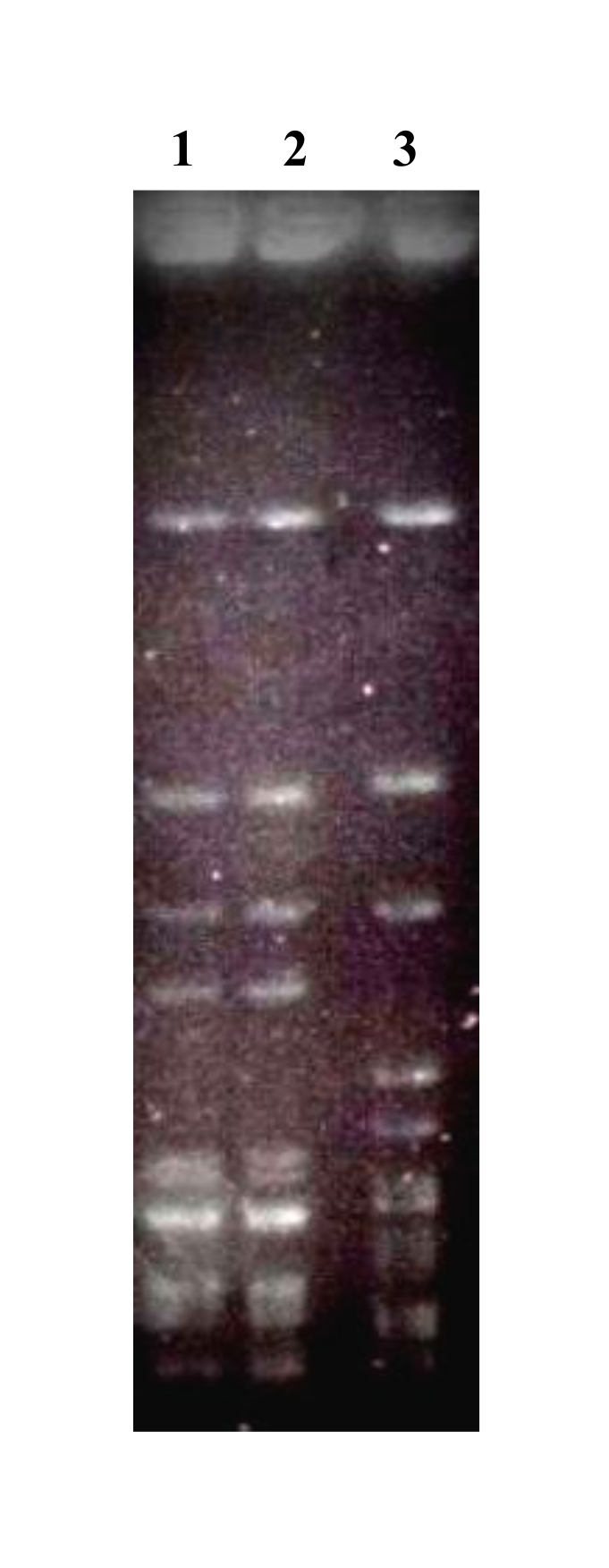

Supplement: Figure S1 — PFGE patterns of SmaI-digested genomic DNA of L. monocytogenes strains. Lanes: 1, BM4715; 2, BM4716; 3, EGD-e. (TIF) [file pone.0106340.s001.tif]
