# Supplementary material for: Overexpression of the Novel MATE Fluoroquinolone Efflux Pump FepA in Listeria monocytogenes Is Driven by Inactivation of Its Local Repressor FepR
Source: PLoS One. 2014 Sep 4;9(9):e106340. doi: 10.1371/journal.pone.0106340 (PMC4154695; doi:10.1371/journal.pone.0106340)
Supplement: Table S1 — Deoxynucleotide primers used in the study. (DOCX) [file pone.0106340.s002.docx]

**Table S1**. Deoxynucleotide primers used in the study.

| Primer^a^ | Sequence (5’ to 3’)^b^ |
| --- | --- |
| PCR-sequencing of QRDRs | |
| Lmo-gyrA-F | AGTGTAATTGTTGCCCG |
| Lmo-gyrA-R | ATATCGCCATCAACCGA |
| Lmo-gyrB-F | AAGCGCGCGCGTGAAGT |
| Lmo-gyrB-R | CGAGATTTAGAAACGTC |
| Lmo-parC-F | GAACGTGCGCTTCCAGC |
| Lmo-parC-R | GTTGCATAACCAGCGGA |
| Lmo-parE-F | GGAAAATTAACGCCAGC |
| Lmo-parE-R | TCGGTCATGATAACTAC |
|  |  |
| qRT-PCR | |
| Lmo-fepA-F | CCGCGATTCAACCACTTATT |
| Lmo-fepA-R | GAGACCAACGAGCAAAAAGC |
| Lmo-lde-F | GGGGTGCTTTCTAACCGGAA |
| Lmo-lde-R | GAGTGCAATCCCTGACCACA |
| Lmo-mdrL-F | TTTGTGCGTTGGTTAGGTGC |
| Lmo-mdrL-R | TGCCTGCCAAACAAATGACG |
| Lmo-blgA-F | AGCCGTTCACCTCATTCGTT |
| Lmo-blgA-R | ACAGGAAAGGAAGCAGAGCC |
|  |  |
| RACE | |
| Lmo-fepRA-GSP-R1 | TAATATCTGCTTGGACCACT |
| Lmo-fepRA-GSP-R2 | ATAGGCCTCGTCCGTATCGT |
| Lmo-fepRA-GSP-R3 | TTCGCGTCCTTCATAATGGAT |
|  |  |
| Mapping of *fepRA* operon | |
| P1-F | TTGCGACTGCTGAATTTGATCC |
| P1-R | GTTTCTTCAGCCCCACAACC |
| P2-F | AACGGTCGTCAGTTTTTGCG |
| P2-R | CTGGGTCGTTCACGTACACA |
| P2-F* | TGTGTACGTGAACGACCCAG |
| P3-F | TTTTCCTTTGCGATCGGGGA |
| P3-R | CACCAGCAGTCCCAGCAATA |
|  |  |
| Deletion of *fepR* |  |
| Lmo-fepR-F1-EcoRI | CGGGAATTCCCAATACGTAGCAAAGATTTA |
| Lmo-fepR-R1 | CTACGATAACTGCGTCGAAGTTTTGCCCGGTAG |
| Lmo-fepR-F2 | ACGCAGTTATCGTAGACATT |
| Lmo-fepR-R2-EcoRI | CGGGAATTCAGGATATTCATATACTCGAGC |

^a^Lmo, *Listeria monocytogenes*; F, forward primer; R, reverse primer; GSP, gene-specific primer.

^b^Restriction sites EcoRI are underlined.

^c^QRDR, quinolone-resistance determining region; RACE, rapid amplification of cDNA ends.
